# Charting Wellness in India: Piloting the iTHRIVE’s Functional Nutrition Approach to Improve Glycaemic and Inflammatory Parameters in Prediabetes and Type 2 Diabetes Mellitus

**DOI:** 10.7759/cureus.63744

**Published:** 2024-07-03

**Authors:** Mugdha Pradhan, Radhika Hedaoo, Anitta Joseph, Ria Jain

**Affiliations:** 1 Nutrition, ThriveTribe Wellness Solutions Pvt Ltd., Pune, IND; 2 Nutrition, Symbiosis School of Culinary Arts, Symbiosis International (Deemed University), Pune, IND

**Keywords:** inflammation, prediabetes, functional nutrition, type 2 diabetes mellitus, insulin resistance

## Abstract

Introduction

Type 2 diabetes mellitus (T2DM) is characterized by elevation of blood glucose levels due to underlying insulin resistance and inflammation. Multiple modifiable risk factors such as unhealthy dietary habits, physical inactivity, obesity, smoking and psychological stress contribute to T2DM. We investigated the efficacy of a comprehensive functional nutrition approach aimed at mitigating T2DM using the iTHRIVE approach which encompassed anti-inflammatory and elimination diets, micronutrient supplements, physical activity, stress management and environmental modifications through a pre-post study design. The research assessed changes in blood glucose and inflammatory markers following the implementation of the functional nutrition program.

Methods

A prospective pre-post intervention pilot study was conducted at ThriveTribe Wellness Solutions Pvt Ltd. (iTHRIVE), where 50 study participants from urban areas of Pune city, India (n=25 each group) were recruited voluntarily in the age group of 20-60 years. The participants were subjected to 90 days of the iTHRIVE functional nutrition approach which consisted of eliminating certain inflammatory foods and adding a combination of nutritious organic foods, adding dietary supplements like magnesium, vitamin D, alpha lipoic acid, chromium picolinate, berberine and biogymnema, physical activities like resistance training, stress reduction techniques like meditation and deep breathing exercises along with environmental changes. The blood parameters like fasting blood glucose, postprandial blood glucose, glycated haemoglobin (HbA1C), fasting serum insulin, post-prandial serum insulin, high sensitivity C-reactive protein (hs-CRP), erythrocyte sedimentation rate (ESR), vitamin D, body weight and waist circumference were measured before and after the intervention. The changes were statistically analyzed using a paired t-test.

Results

The mean age of the participants was found to be 43.76±10.58 years. Around 68% of the participants were prediabetic (HbA1c: 5.7-6.4%) and 32% had T2DM (HbA1c ≥6.5%). A significant reduction was observed in the average HbA1c (13.75% reduction, p<0.0001), average post-prandial blood glucose levels (14.51% reduction, p<0.048), average post-prandial serum insulin (34.31% reduction, p<0.017) and average ESR levels (34.51% reduction, p<0.006). The hs-CRP levels were reduced by 6.6%, but not statistically significant. The average body weight of the participants dropped from 78.59±15.18 kg to 75.20±14.20 kg with a mean loss of 2.91 kg (p<0.05) whereas the waist circumference decreased from 37.54±5.09 to 35.97±4.74 inches with an average loss of 1.19 inches (p<0.0004).

Conclusions

Following the intervention, several health indicators indicated significant improvements. Particularly, there was a significant drop in HbA1c levels, suggesting better long-term blood glucose control. Blood glucose and serum insulin levels after a meal dropped significantly, indicating enhanced insulin sensitivity. There was a decrease in systemic inflammation as evidenced by the decrease in ESR levels. These results imply that the iTHRIVE functional nutrition approach used in this investigation might be beneficial for enhancing glycemic control and insulin sensitivity, along with reducing inflammatory markers in people with prediabetes and T2DM. Larger sample sizes and longer periods of monitoring would be useful in subsequent research to validate and build on these encouraging findings.

## Introduction

The International Diabetes Federation (IDF) has estimated that 77 million individuals had diabetes in India in 2019 and is expected to cross 134 million by 2045 [1]. The majority of the cases are type 2 diabetes mellitus (T2DM), a complex condition characterised by a deficiency in the insulin secretion by β-cells of the pancreas, tissue insulin resistance (IR) along with an insufficient compensatory insulin secretory response [2]. IR can be defined as an attenuated response of tissues to normal or elevated physiological levels of insulin [2]. People who are at risk for T2DM initially have a state of IR which is compensated with hypersecretion of insulin by the β-cells of the pancreas. Eventually, the pancreas becomes unable to cope up with the insulin secretion which ultimately leads to β-cell dysfunction [3]. Moreover, chronic systemic inflammation caused by unhealthy diet patterns, obesity, physical inactivity and psychological stress activates the innate immune system and causes the secretion of pro-inflammatory cytokines [3]. Prolonged exposure of pro-inflammatory mediators stimulates the activation of cytokine signalling proteins which block the activation of insulin signalling receptors in β-cells of the pancreas. This leads to dysregulation in glucose homeostasis and causes a consistent elevation in blood glucose levels. Such a hyperglycemic state creates oxidative stress in the body, ultimately damaging the pancreatic islet cells and inhibiting insulin gene expression [3].

Numerous factors like genetics, metabolic, lifestyle and environmental factors interact with each other and contribute to T2DM development [4]. Genetic predisposition is an important contributing factor for T2DM [5]. The prevalence of T2DM varies depending on the ethnicity. Studies have shown higher incidence rates in Asians as compared with the American population. Lifestyle, socioeconomic factors and gene-environmental interactions have been thought to influence T2DM development [4].

While individuals can be predisposed to T2DM on account of non-modifiable risk factors such as ethnicity, family history or genetic predisposition, evidence from epidemiological studies point out that T2DM condition could be improved by working on major modifiable risk factors like an unhealthy diet, lack of physical activity, obesity, smoking and psychological stress [6,7].

Unhealthy diet patterns along with obesity and physical inactivity pose the strongest risk factors for IR and T2DM [2]. A recent meta-analysis has shown that both moderate and high consumption of ultra-processed foods increases the risk of T2DM by 12% and 31% respectively [8]. It was also observed in a prospective cohort study from nine countries (Asian and Western countries) that long-term consumption of sugar-sweetened beverages (also called sugary drinks or soft drinks) increases the risk of T2DM by 29% [9]. To date, various dietary patterns have been considered beneficial for T2DM. High-protein and low-carbohydrate dietary model has been found to reduce plasma insulin levels possibly due to the reduction in glucose load [10]. A ketogenic diet with high-fat, moderate-protein and low-carbohydrate has been shown to decrease inflammation in the pancreas and enhance insulin sensitivity [10]. Mediterranean diet which is a low-carb diet, rich in monounsaturated and polyunsaturated fatty acids (PUFA) and antioxidants like polyphenols, has been found to combat inflammation and increase insulin sensitivity [10]. Such dietary models mostly consist of a combination of nutrient-dense foods leading to lowered inflammation, reduced glucose load and essential nutritional support to the body.

One such dietary model involves a gluten-free (GF) diet. A diet containing wheat flour has been linked to a higher occurrence of diabetes mellitus in animal studies [11]. In non-diabetic mice, consumption of a wheat-based diet increased the incidence of diabetes mellitus and the severity of insulitis. Conversely, feeding these mice a diet based on hydrolyzed casein resulted in a reduced incidence of diabetes. Moreover, diets that exclude wheat and barley proteins have been associated with a reduced incidence and delayed onset of diabetes [11].

There has been an increasing amount of evidence for the role of gluten and gut health in T2DM [12,13]. Studies show that the consumption of foods such as wheat and rye containing gluten affects the gut microbiota adversely leading to damage of the intestinal lining, causing inflammation and aggravating IR. Moreover, proline, glutamine and hydrophobic amino acids are the primary amino acid components of gluten, which hinders gluten from being entirely broken down by pancreatic, stomach, and brush-border enzymes [12,13]. Additionally, the gluten peptides like gliadin present in wheat could cross the intestinal barriers affecting the pancreatic beta cells directly [14].

It was evidenced that such dietary practices have a significant impact on glycemia and gut flora [15]. A gluten-containing diet was observed to increase the prevalence of hyperglycemia in non-obese diabetic mice, whereas a GF diet lowered it. Moreover, it was shown that the faecal microbiota varied depending on the diet. In a gluten-containing diet, *Bifidobacterium *spp. was more common, whereas in a GF diet, *Akkermansia *spp. was more common [15].

A sedentary lifestyle is a major precipitating factor of diabetes mellitus. A high correlation of physical inactivity has been linked with T2DM incidence, since it can also contribute to obesity [16]. Additionally, psychological stress is also considered as a prominent factor in T2DM [17]. Stress can lead to the stimulation of hypothalamus-pituitary-adrenal gland (HPA), thus causing increased cortisol levels. Prolonged elevations in cortisol can lead to blood sugar dysregulation through gluconeogenesis [17]. Moreover, recent studies are unveiling the effects of environmental factors like exposure to endocrine-disrupting chemicals (EDCs) [18,19], exposure to artificial lights at night and associated circadian rhythm disruption [20,21], etc. in causing metabolic disturbances. EDCs are exogenous chemicals, or a mixture of chemicals, that can interfere with any aspect of hormone action [18]. Substances like household chemicals, fragrances, lotions, soaps, etc. can act as sources of EDCs. It has been found that EDCs like bisphenols, phthalates, parabens and heavy metals could potentially alter the β-cell physiology and cause IR [22]. Industrialisation and electrification in the modern world have led to an increased indoor time and a minimal outdoor time and the resultant sleep and circadian rhythm disruption has been found to acutely impair glucose tolerance [20,23]. Instances of T2DM incidences in obstructive sleep apnea patients have corroborated the significance of sleep and circadian rhythm in T2DM [24].

However, the effectiveness of integrating these dietary patterns with other lifestyle factors such as physical activity, stress management and environmental influences such as digital detox and avoidance of use of plastics remains insufficiently studied within the Indian population. We hypothesized that improving factors such as diet, physical activity, nutritional deficiencies, stress and environmental aspects may lead to an improvement in the glycaemic markers and IR. Thus the aim of the study was to observe the apparent changes in the glycaemic and inflammatory parameters before and after the dietary and lifestyle intervention and thereby assess the efficacy of this approach.

## Materials and methods

Study participants

This was a pilot pre-post study conducted by ThriveTribe Wellness Solutions Pvt Ltd., also known as iTHRIVE, which is located in Pune city. The participants approached iTHRIVE through its advertisements on social media and referral programs. The enrolment commenced in March 2021 and 50 participants were selected for the study. The participants were purposefully selected to have an equal number of males and females. An informed written consent was obtained from the participants prior to the commencement of the program. The ethical approval for conducting the study was obtained from the Institutional Ethics Committee (IEC) (SIU/IEC/292) of Symbiosis International University, Pune. The inclusion criteria were as follows: those between the ages of 20 years to 60 years and prediabetics with HbA1C levels equal to or greater than 5.7%. As per the American Diabetes Association (ADA), HbA1C levels ranging from 5.7% to 6.4% can be classified as prediabetes and diabetics with HbA1C levels equal to or greater than 6.5% are classified as diabetics. The exclusion criteria were as follows: individuals with chronic diabetic complications or other co-morbidities and diseases, pregnant females, lactating mothers, teenagers, individuals with genetic diseases, individuals with type 1 diabetes and other autoimmune disorders and individuals following any Ayurveda treatments or medications. Additionally, those participants who were on medications were allowed to continue them during the course of the study.

Functional nutrition approach

**Figure 1:**
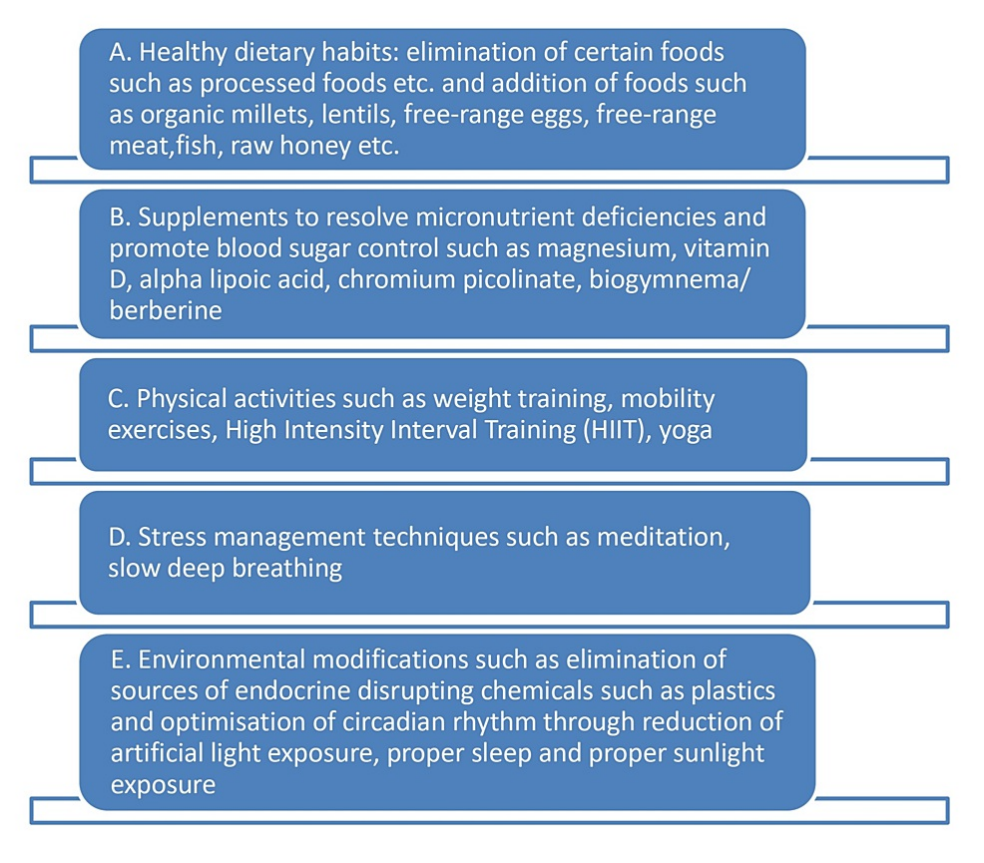
Outline of iTHRIVE functional nutrition approach for improving glycaemic and inflammatory parameters in prediabetes and type 2 diabetes mellitus

The study timeline for the 50 participants spanned an average period of 90 days starting from June 2021 after obtaining IEC approval. At the beginning of the program, three-day diet recalls (two weekdays and one-weekend recall) were obtained from the study participants along with their other aspects such as exercise, stress, sleep, sunlight/daylight exposure, screen time, etc. through an online consultation call. Following the preliminary analysis, the participants were briefed about the study program. The study design involved a holistic functional approach (Figure 1) which comprises addition and elimination diets, supplements for correcting deficiencies, physical activity, stress management modalities and environmental changes such as digital detox, avoidance of endocrine disruptors like plastics, etc.

The first component of this approach was the incorporation of healthy dietary patterns which involved an elimination of pre-decided foods and ingredients followed by the addition of certain food sources. As part of the elimination, food sources such as ultra-processed packaged foods, sugary beverages, alcohol, etc. were excluded from the diet. According to the US National Survey 2016 [25], ultra-processed food is defined as industrial formulations with five or more ingredients such as hydrolyzed protein, modified starches, hydrogenated oils, and additives such as colourants, non-sugar sweeteners, etc. Refined sugar, sweetened beverages, sports drinks, cakes, cookies, soda, candy, ice cream, flavoured yoghurts and snack bars were eliminated further. Refined seed oils were also eliminated on account of lipid peroxidation and subsequent production of free radicals which could activate inflammatory pathways [26].

The participants were encouraged to use unprocessed foods or minimally processed foods (these are unprocessed foods that undergo industrial processes such as drying, crushing, grinding, etc.) as a part of the dietary modification. They were also advised to eliminate gluten-rich foods such as wheat, oats, rye, refined wheat flour, semolina, vermicelli, barley and other wheat products. Millets and rice were the recommended alternatives.

Other than the three milk products - cow’s ghee, butter and buttermilk - all other milk and milk products were avoided on account of genetic modification of cows and hormonal injections for milk production [27]. Such high estrogenic milk might cause conditions such as acne, diabetes, IR [28], obesity, and hormonal imbalances. Consumption of walnuts was specifically encouraged in this dietary protocol due to its uniquely high PUFA content, which has been evidenced in reducing blood sugar levels [29].

While positive correlations were observed for fruit juices and fruit drinks with T2DM, evidence suggests a reduction in the risk of T2DM with the intake of fruits, especially berries, apples, grapes and yellow vegetables [30]. Thus, fresh fruits and starchy vegetables were included as healthy sources of carbohydrates.

For cooking purposes, organic or grass-fed cow’s ghee, organic grass-fed or homemade butter or organic cold-pressed (virgin) coconut oil was recommended and refined seed oils were eliminated. Cooking methods such as deep frying, commercially grilled foods which use high-calorie marinades, reused oils for grilling pan frying, etc. were advised to be avoided considering the generation of high transfats, formation of heterocyclic amines (HCAs) and polycyclic aromatic hydrocarbons (PAHs) and high sodium content [31].

Studies show a higher protein intake (around 1.2 g per kg) to be beneficial for improving IR [32]. Unprocessed grass-fed organic red meat like lamb, ribs, organ meats like liver, free-range poultry and free-range organic eggs were included in the dietary approach as higher bioavailable protein sources. Seafood, both white fish and oily fish, was recommended and has been shown to be beneficial in diabetes which may be attributed to the omega three fatty acids [30].

For ease of acquisition, a list of vendors specifically selling grass-fed and organic products was shared with the participants. Considering the above aspects, the iTHRIVE diet plan was shared with the participants (Table 1) along with useful food recipes.

**Table 1:** iTHRIVE diet chart for the management of prediabetes and T2DM EPA: eicosapentaenoic acid; DHA: docosahexaenoic acid; T2DM: type 2 diabetes mellitus

Time	Meal components	Rationale/justification
Morning (empty stomach)	To start the day, a glass of fresh lemon water was prepared by squeezing the juice of one lemon into a glass of water and adding a pinch of cinnamon powder. Following this, 5-6 tulsi (holy basil) leaves were chewed and swallowed, ensuring they were thoroughly chewed before swallowing. After a half-hour interval, one teaspoon of fenugreek seeds that had been soaked in water overnight for six to eight hours was consumed. The soaking water was discarded before consumption. If needed, a half glass of plain water was used to facilitate the proper chewing and swallowing of the fenugreek seeds.	Lemon is rich in Vitamin C and has antioxidant and anti-inflammatory properties [33]. Cinnamon has an insulin-potentiating effect (however, evidence is inconclusive) [34]. Basil leaves have eugenol which exerts a hypoglycemic effect [33]. Fenugreek suppresses inflammation and has antidiabetic properties [34].
Exercise	Resistance exercises or running/brisk walking/yoga for 30-45 minutes are advised.	Exercise i­­mproves glycemic control, increases insulin sensitivity [17].
Post-workout meal	Fifteen to twenty minutes after the workout, either of the following was consumed: two or three eggs, which were either boiled, scrambled, or made into an omelette, or, for vegetarians, one scoop of whey protein.	Protein intake soon after the exercise increases recovery and helps in skeletal muscle building. Active skeletal muscles increase glucose uptake and exert glycemic control [17].
Breakfast	One hour after the workout, breakfast was consumed with the following options: The first option included a millet and legume-based breakfast, which could consist of either two besan cheelas, two green moong dosas, three to four idlis, or one to two dosas. The side dish for this option was two tablespoons of either coconut, mint, or coriander chutney. Alternatively, oats upma (using steel-cut or rolled oats) or millets upma were other options. Following the meal, one half or one whole walnut, which had been soaked in water for seven to eight hours, was consumed.	Due to the high fiber content and resistant starch, millets and legumes slows the glucose release into bloodstream and exert glycemic control [35]. Protein intake in the morning leads to reduction in insulin spikes and achieve greater blood sugar control. Walnuts aid in restoring insulin sensitivity and possess hypoglycemic properties [29].
Lunch	The meal consisted of a bowl of salad made with carrots, tomatoes, cucumbers, lettuce, and onions. Alternatively, salads made with sprouts, which were either cooked or steamed, were also an option. For the main course, vegetarians consumed one to two bowls (150-250 mL) of thick dal (any type of dal was used). Additionally, a half bowl (30 grams) of cooked rice or whole grains was consumed. Alternatively, half a bhakri or roti made with 15-20 grams of raw millet flours such as proso, kodo, barnyard millet, sorghum, or bajra, or rice was an option. Non-vegetarians opted for 150 to 200 grams of free-range chicken or 150 to 200 grams of seafood like butterfish, anchovies, small mackerel, shrimps, prawns, or herring. Finally, two to three raw garlic pods were added to the meal. Optionally, a glass of buttermilk made from A2 milk curd or coconut milk curd was included.	Vegetable intake at the start of the meal followed by protein and carbs leads to a controlled release of glucose in bloodstream. Omega 3 fatty acids like EPA and DHA in seafood have anti-inflammatory properties. Garlic improves digestion and is a potent antioxidant [30]. Buttermilk/curd acts as probiotics and influences glucose absorption. Polyphenols in vegetables and fruits act as antioxidant and anti-inflammatory compounds [30].
Evening tea and snacks (if needed)	Fluids such as ginger lemon tea or turmeric and pepper herbal tea were advised. Use of organic honey as sweetener in limited quantities was permitted. Coconut water was advised. Snack options such as protein cookies or sorghum cookies or one bowl of sprouts/steamed sprouts salad or sprouts dhokla or lentil soup were advised. Alternatively, seasonal low glycemic index fruits or one bowl of mixed fruit salad made from citrus fruits was advised [36].	Herbal infusions have anti-inflammatory and antioxidant effects. Sprouts are easy on the gut and rich in proteins. Fruits contain polyphenol compounds having anti-oxidant effects. Coconut water is a rich source of electrolytes [30].
Dinner	The first option included a vegetable salad made with cucumber, carrot, onion, tomato, and radish. One teaspoon of fresh lemon juice was sprinkled on the salad before consumption. The second option included two boiled eggs, scrambled eggs, or an egg omelette. Additionally, twice a week, 150 to 200 grams of organic free-range chicken or 150 to 200 grams of seafood such as butterfish, anchovies, small mackerel, shrimps, prawns, or herring were consumed. Grains were avoided for dinner.	Polyphenols in vegetables confer antioxidant properties. Protein and fats promote satiety. Omega 3 fatty acids like EPA and DHA in seafood have anti-inflammatory properties. Grains are avoided to prevent insulin spikes close to bedtime so that it doesn’t interfere with sleep [30].

The second component involved correcting micronutrient deficiencies such as magnesium and vitamin D. Supplementation with essential micronutrients in recommended dosages was found to resolve nutritional deficiencies [37]. Magnesium deficiency is found to be associated with metabolic disorders like diabetes. Magnesium ion plays a major role in carbohydrate metabolism and insulin action [38]. Vitamin D deficiency alters insulin secretion whereas its replenishment improves glycaemia and IR [38]. Magnesium glycinate was given in powdered form and taken one scoop before bedtime. One scoop would contain 400 mg of elemental magnesium. Vitamin D dosages were adjusted as per the individual levels of the participants and were monitored with blood tests. Both the supplements were given under medical supervision and were checked for toxicity.

Additionally, blood sugar-regulating supplements such as chromium picolinate (400 mcg once a day) and berberine (500 mg twice a day) or biogymnema (customized dosage) and alpha lipoic acid (300 mg once a day) were included. The participants were made aware of the benefits of supplementing with these and were given under medical supervision.

The third component involved adequate physical activity. Daily or routine physical activity spanning for a period of 30-45 minutes like brisk walking, running, swimming or weight training was also recommended in the approach. Flexibility and mobility exercises were also advised. These were recommended by the in-house sports nutritionists and were individualised according to the participants' needs.

The fourth component comprised various stress-reducing modalities like meditation and breathwork for a period of 15 minutes. The assigned wellness experts would explain the methods and benefits of practising meditation and deep slow breathing. Pre-recorded audios were shared for guided meditation and breathing techniques.

The fifth component involved various environmental modifications such as the elimination of sources of EDCs, reduction of artificial light exposure and optimisation of circadian rhythm. During the consultation calls, the participants were counselled on the adverse effects of plastics and artificial light exposure and its effects on beta cell function and insulin secretion. The participants were advised to replace the plastic containers for food storage or water storage with glass or steel materials so as to reduce the exposure to endocrine disruptors. It was also advised to reduce exogenous toxin exposure through measures like swapping chemical-based soaps, shampoos, perfumes, detergents and other personal care products for natural or organic alternatives. Substitutes like reetha and shikakai were recommended for washing hair. For circadian rhythm optimisation, the participants were advised to reduce the artificial light exposure from screens, especially at night time or close to bedtime. They were directed to install blue light filters in their screens and a digital detox of two to three hours before sleeping was recommended. Moreover, moderate sun exposure from 11 am to 4 pm (preferably 11 am-1 pm), for 20-30 minutes [39], was also advised.

The intervention lasted for an average period of 90 days starting from June 2021, for each participant. The participants had real-time access to the team of nutritionists through Whatsapp, where they were able to converse with them regarding the interventions. The weekly follow-ups were done through online video calls conducted by the team of functional nutritionists at iTHRIVE, Pune. The tracking of meals, water intake, supplements, physical activity, mediation practices and sleep time was done for the entire study duration. During the weekly consultation calls with the participants, the nutritionists would track the improvements and perform dose adjustments of the supplements wherever necessary. To measure the effectiveness of this approach, various blood biomarkers for monitoring IR and inflammation were used. Parameters consisted of diabetes markers such as fasting blood glucose, post prandial blood glucose, HbA1c, fasting serum insulin, post prandial serum insulin and inflammation markers such as high-sensitivity C-reactive protein (hs-CRP) and erythrocyte sedimentation rate (ESR). The blood samples were collected before (at the time of enrollment) and after the intervention (preferably within one month) through the National Accreditation Board for Testing and Calibration Laboratories (NABL)-accredited partnered lab.

Statistical analysis

The data was analyzed using Microsoft Excel version 2010 (Microsoft® Corp., Redmond, WA, USA) as well as using built-in functions on Google Sheets for the entire study duration. Descriptive statistics were used to summarize the clinical characteristics of the patient blood sample. The normality of the data was assessed using the Shapiro-Wilk test. A paired t-test was used to determine the mean difference in biomarkers pre-intervention and post-intervention. A p-value of less than 0.05 was considered statistically significant at a confidence level of 95%.

## Results

The mean age of the entire study participants was 43.76±10.58 years; the mean age of female participants was 42.2±11.50 years and that of male participants was 45.32±9.60 years. There were no dropouts but two participants failed to report the post-protocol blood analysis. During the preliminary consultation call, it was revealed that the participants had unhealthy eating patterns, followed minimal to no physical activity and were overweight as indicated by their average BMI of 28.5 kg/m^2^. During the three-day diet recall, the participants revealed that gluten-rich foods, sugars, refined vegetable seed oils and hydrogenated fats, ultra-processed foods, and milk and milk products constituted the major portions of their diet. The participants also reported spending the majority of time indoors without proper sunlight/daylight exposure and it was reflected in their insufficient average vitamin D levels measured as 27.74 ng/mL [40]. Additionally, they also mentioned sleeping less than seven hours a day, interrupted sleep patterns at night and usage of devices such as TV and smartphones close to bedtime.

The blood parameters such as fasting blood glucose, postprandial blood glucose, HbA1c, fasting serum insulin, postprandial serum insulin and inflammation markers such as hs-CRP and ESR were measured before and after the intervention (Table 2). Fasting and postprandial plasma glucose levels provide a direct measure of the glycemic status [41]. Serum insulin level (fasting or postprandial) is an independent biomarker for the risk of IR [42]. HbA1c gives the average blood sugar level over the past three months [41]. Increased levels of hs-CRP and ESR are better markers of systemic inflammation which is related to metabolic syndrome like diabetes [41].

**Table 2:** Blood parameters before and after the intervention. The statistical analysis was done using a paired t-test. All values are expressed as mean ± standard deviation (n=50). HbA1C: glycated haemoglobin; hs-CRP: high sensitivity C-reactive protein; ESR: erythrocyte sedimentation rate; mg/dL: milligrams per deciliter; μIU/mL: microinternational units per millilitre; ng/mL: nanograms per millilitre; mg/L: milligrams per litre; mm/hr: millimetres per hour

Parameters with units	Before intervention	After intervention	p-value
Fasting blood glucose (mg/dL)	109.83 ± 40.71	103.13 ± 25.89	0.0892
Postprandial blood glucose (mg/dL)	140.35 ± 74.47	121.37 ± 39.78	0.0483
HbA1C (%)	6.92 ± 1.95	6.03 ± 0.68	0.0001
Fasting serum insulin (μIU/mL)	16.036 ± 23.02	11.79 ± 9.26	0.1176
Postprandial serum insulin (μIU/mL)	59.93 ± 61.92	42.38 ± 45.87	0.0178
hs-CRP (mg/L)	4.93 ± 5.41	4.62 ± 7.45	0.3887
ESR (mm/hr)	18.96 ± 15.78	13.38 ± 10.28	0.0063
Serum 25-hydroxyvitamin D (ng/mL)	27.74 ± 16.18	31.03 ± 11.91	0.0503
Waist circumference (inches)	37.54 ± 5.09	35.97 ± 4.74	0.0004
Body weight (Kg)	78.59 ± 15.18	75.20 ± 14.20	<0.05

Pre-intervention, it was observed that 68% of the participants had HbA1C levels ranging from 5.7% to 6.4% (prediabetes) while the remaining 32% had HbA1C levels equal to or above 6.5% (diabetes). The average body weight of the participants before the intervention was 77.6±16.02 kg and the waist circumference was 37.57±5.10 inches.

Throughout the study period, daily tracking of the dietary habits and online follow-up calls revealed that the participants were able to follow most of the dietary modifications. Participants reported positive gut health and improved satiety with an elimination diet and including protein-rich sources as part of the addition diet. They reported regular administration of dietary supplements and blood sugar-regulating supplements under medical supervision. For those who experienced gastric discomfort with magnesium, the doses were reduced at the beginning and built up to the required dose with time. Additionally, the participants reported a boost in their energy levels while incorporating physical activities into their routines. Screen time was reduced by half an hour as mentioned by the participants in their follow-up calls. They experienced uninterrupted sleep patterns, especially women and reported that they were able to sleep an extra hour than usual. They also mentioned quick onset of sleep at night with sunlight exposure for at least 20 minutes and decreasing artificial light/screen exposure at least half an hour before bedtime. They reported calmness and relaxation with meditation practices and breathing exercises for about 15 minutes a day. Participants self-reported their genuine efforts in swapping plastic food containers, non-stick pans, chemical-based personal care products, etc. with alternatives such as steel food containers, stainless steel or cast iron pans, natural personal care products, etc.

The blood analysis performed post-intervention corroborated the above-mentioned improvements. Following the blood analysis, it was observed that the average HbA1c was reduced by 0.85% (p<0.0001), average postprandial fasting blood glucose levels were reduced by 16.7 mg/dL (p<0.048) and average postprandial serum insulin dropped by 14.73 μIU/mL (p<0.017). The average ESR levels showed a difference of 5.36 mm/hr (p<0.006). Additionally, the average body weight of the participants dropped from 78.59±15.18 kg to 75.20±14.20 kg with a mean loss of 2.91 kg (p<0.05) whereas the waist circumference decreased from 37.54±5.09 inches to 35.97±4.74 inches with an average loss of 1.19 inches (p<0.0004). Moreover, the results showed a significant increase in vitamin D levels from 27.74 to 31.03 ng/mL (p<0.05) (Table 2).

In the pre-diabetes (HbA1c levels: 5.7% to 6.4%) group, HbA1c levels dropped from 6.05±0.58% to 5.75±0.41% with an average drop of 0.29% (p<0.05). In contrast, in the group with T2DM (HbA1c equal to or greater than 6.5%), the HbA1c levels dropped from 8.83±2.5% to 6.64±0.75% with an average drop of 2.05% (p<0.05). Thus, the T2DM group showed significant improvements in HbA1c levels as compared to the prediabetes group.

However, significant changes were observed in the average body weight and waist circumference pre- and post-intervention in the prediabetes group as compared with the T2DM group (p>0.05). The average waist circumference changed from 36.94±4.94 inches to 35.20±4.29 inches with a mean loss of 1.43 inches (p<0.05). The average body weight also dropped from 78.04±16.27 kg to 74.77±14.24 kg with a mean loss of 3.71 kg (p<0.05). In the T2DM group, the mean weight loss was 1.20 kg and the mean loss in waist circumference was 0.66 inches which was not statistically significant.

## Discussion

The urban Indian population has seen a demographic shift accompanied by an upsurge in sedentary occupations leading to minimal physical activity. In India, type 2 diabetes, a condition influenced by both genetic predisposition and sedentary lifestyle choices and a high carbohydrate and highly processed diet, has become increasingly prevalent in urban Indian settings, replicating the global trends in major metropolitan cities of the world [43].

The iTHRIVE functional nutrition approach focuses on a millet-based, low carbohydrate, organic, GF diet with plenty of vegetables and elimination of processed foods, supplementation, physical activities, mental health and environmental changes targeted at reducing inflammation in the body, thereby improving IR. Inflammation is the foremost root cause which drives IR and T2DM [3]. Our functional nutrition approach was followed to observe the changes in the biochemical parameters and thus, assess the improvements in glycaemic and inflammatory markers.

A similar study was conducted in England where participants were continuously advised to a low carbohydrate diet over a period of 23 months bringing a weight loss of 8.3 kg and a decrease in HbA1c by 1.6% in the T2DM group. In the prediabetes group, the drop in HbA1c was 0.5% [44]. Another retrospective study reported significant changes in HbA1c, fasting blood sugar and body weight following a 90-day personalized intervention program which consists of complex carbohydrates, low glycaemic index, glycaemic load, and gut-healthy foods followed by regular exercise and lifestyle modification. The study reported the mean changes in HbA1c, fasting blood sugar and body weight as 1.9±1.5%, 62.2±51.8 mg/dL and 2.8±1.6 kg respectively [45].

A single-arm real-world observation study denotes that a millet-based intervention diet can cause a significant decline in blood glucose, HbA1c, oxidative stress and pro-inflammatory cytokines [35]. A meta-analysis of randomised controlled experimental trials in people with and without diabetes has observed that pulses like chickpeas, beans, peas, lentils, etc. provide a good source of slowly digestible carbohydrates and fibre. It was also observed that the pulses like mung beans when sprouted, exhibited improved anti-inflammatory, antioxidant and anti-diabetic effects [46]. Moreover, intake of organic raw honey has been found to positively influence body weight and glycemic markers [47].

A review by Mir et al. has suggested the abundance of conjugated linoleic acid, omega-3 fatty acids, β-carotene, α-tocopherol (vitamin E) and antioxidant enzymes in grass-fed cow’s ghee and significant amounts of medium chain fatty acids like lauric acid in virgin coconut oil that confers antioxidant, antithrombotic and anti-inflammatory properties [48]. Another review discusses that coconut oil possesses antioxidants which improve insulin response in hyperglycemic patients and thereby reduce IR [49].

A prospective cohort study in the US has evidence that such unprocessed red meat was not associated with diabetes incidence, but processed meats were positively associated with diabetes incidence [50]. Seafood, both white fish and oily fish, was shown to be beneficial in diabetes which may be attributed to the effect of omega three fatty acids present in them [30].

A double-blinded randomized study has reported decreased body weight and increased insulin sensitivity in T2DM patients after taking chromium picolinate supplements [51]. A study done in 2006 reported improved insulin sensitivity in T2DM patients after oral administration of alpha-lipoic acid 600 mg twice daily for a period of four weeks. Alpha lipoic acid was found to decrease the oxidative stress in cells and thereby has a therapeutic use in diabetes [52]. Studies have shown that intake of biogymnema extracts exerts a hypoglycemic effect through an increase in plasma active glucagon-like peptide (GLP-1) levels [53]. A systematic review and meta-analysis of randomized controlled trials have reported strong evidence for the effectiveness of berberine in lowering blood glucose levels [54]. Since the iTHRIVE approach involved correcting the nutrient deficiencies with magnesium and vitamin D along with chromium picolinate, alpha lipoic acid and biogymnema/berberine, it would have led to an overall increase in insulin sensitivity and reduction in inflammation.

Physical activity has been widely considered as an integral part of T2DM intervention [17]. Resistance exercises promote skeletal muscle building and increase glucose uptake receptors, thereby promoting efficient glucose uptake in individuals with T2DM. Aerobic exercises like brisk walking, jogging, swimming and anaerobic exercises like resistance training increase insulin sensitivity [17]. It has also been reported that the addition of meditation and slow deep breathing techniques with the exercise regimen could better improve glycemic control, body weight, blood pressure and waist circumference [17]. It also contributes to reduced stress and anxiety, improved social relationships and greater adherence to positive lifestyle and self-care. Consumer products like soaps, shampoos, detergents, personal care products, etc. contain EDCs which can potentially reduce the insulin sensitivity of target cells [9]. Plastic food containers have EDCs like bisphenols which alter the β-cell physiology and cause IR [9]. The elimination of such EDCs becomes indispensable, especially in the context of lifestyle diseases. Moderate sun exposure helps in the alignment of circadian rhythm and regulates glycemic status [8]. Moreover, it increases vitamin D levels, known to influence the insulin sensitivity of cells and regulate glucose metabolism [55]. Excessive blue light exposure at night time disturbs the circadian rhythm of the body. In a diabetic context, it impairs glucose tolerance and causes an increased sugar intake, resulting in IR [56]. A clinical study has reported that wearing blue-light shield eyeglasses brought improvements in fasting blood glucose, IR and sleep quality [57]. The usage of blue blockers or turning screens red would be a practical approach to minimize the detrimental effects of blue light.

A dose-response meta-analysis has shown an increase in the risk for T2DM corresponding to an increase in the intake of processed foods from 10% to 15% [13]. The observed decrease in inflammation indicators, reflected by lower ESR and hs-CRP levels may have been caused, in particular, by the interventions involving dietary adjustments, exercise, stress management and other lifestyle changes. Reduced HbA1c, postprandial glucose and postprandial insulin levels suggest that incorporating high-quality protein and moderate amounts of complex, unprocessed carbs improves blood glucose profiles. The lifestyle and environmental changes might also have accelerated and augmented the entire benefits by promoting holistic healing on all levels; however, further studies are needed to confirm these aspects.

The novelty of this pilot study is the adoption of a holistic perspective for tackling prediabetes and diabetes mellitus through a wellness approach. Additionally, the study is done in an Indian context and the intention was to ensure that the findings and recommendations are relevant, feasible and easily adoptable for people living in India. Since this is a pilot study, it restricts the generalisation of the study results. Considering the protocol to be of a limited duration of 90 days, further follow-up is required to check the sustainability of the protocol for inflammatory parameters and glycemic parameters.

## Conclusions

From the results of our pilot study, it was observed that functional nutrition and lifestyle strategies reduce systemic inflammation and increase cell sensitivity to insulin, which could ultimately lead to improved blood sugar and inflammatory markers in prediabetes and T2DM. The improvements were achieved by implementing healthy dietary habits along with moderate exercise, micronutrient supplementation, stress management and other environmental changes such as digital detox, reduction of toxins from personal care products and household products, etc. Thus, the iTHRIVE functional nutrition approach can be recommended for improving glycemic and inflammatory parameters in T2DM. This study has the potential to be expanded to a larger cohort size with a diverse population. Further research is warranted to decipher the effects of lifestyle and environmental factors and how they affect the overall management of prediabetes and T2DM.
